# Effects of strip-tillage on soil microbial community structure and function in black soil

**DOI:** 10.3389/fmicb.2025.1730920

**Published:** 2025-12-23

**Authors:** Cunxia Yuan, Zhixing Ma, Siyang Liu, Hongli Nie, Guozhong Feng, Shaojie Wang, Shasha Luo

**Affiliations:** 1 Key Laboratory of Sustainable Utilization of Soil Resources in Commodity Grain Base of Jilin Province, Jilin Agricultural University, Changchun, China; 2 State Key Laboratory of Black Soils Conservation and Utilization, Research Center of Mollisols Agroecology, Northeast Institute of Geography and Agroecology, Chinese Academy of Sciences, Changchun, China

**Keywords:** strip tillage, straw belt, maize belt, freeze–thawing, function prediction

## Abstract

**Introduction:**

The spatial heterogeneity introduced by strip tillage (ST; maize belt (ST-M) and straw belt (ST-S)) leads to the pronounced differentiation in soil properties. However, its effects on soil microbial community structure and function remain unclear.

**Methods:**

In this study, amplicon sequencing (Accu16S™ and AccuITS™) was used to investigate the effects of different tillage practices on soil microbial communities.

**Results:**

The results showed that the ST and ST-S treatments significantly increased the Shannon diversity index of microbial communities compared to rotary tillage (RT). Tillage practices also influenced microbial community structure, with fungal communities showing a more pronounced response than bacterial communities. Compared to the RT treatment, the ST-M, ST-S, and ST treatments significantly increased the relative abundance (RA) of Gemmatimonadetes and reduced the RA of Acidobacteria. Additionally, the ST-S and ST treatments significantly enhanced the absolute abundances (AAs) of *Arenimonas* and *Luteolibacter* compared to the RT treatment. Following freeze–thaw events, the ST-M, ST-S, and ST treatments significantly increased the AAs of Latescibacteria, while significantly increasing the AA of *Microvirga* compared to the RT treatment. Furthermore, Mantel test showed that soil bacterial communities were significantly correlated with electric conductivity (EC) and available potassium, while soil fungal communities were significantly correlated with EC and soil organic carbon. Functional prediction revealed that ST significantly promoted nitrification, denitrification, sulfur oxidation, and ectomycorrhizal.

**Disscussion:**

Therefore, strip tillage could improve microbial community diversity and microbial regulation of the N and S cycles in black soil, providing a microbiological perspective for conservation agriculture.

## 1 Introduction

Black soil is facing a severe degradation. The global black soil region covers less than 7% of the world’s land area and is primarily concentrated in the fertile Ukrainian Great Plains, the Mississippi River Basin in the United States, the Northeast Plains of China, and the Pampas grasslands of South America (Li et al., 2025). Despite this limited area, these fertile soils are fundamental to global food security, are often described as the ‘breadbaskets’ of their respective continents. The total area of the black soil region in Northeast China is 1.09 million square kilometers, of which 1.85 × 10^5^ square kilometers are typical cultivation regions (Sui and Dai, 2025). However, under the dual pressures of human activities and climate change, black soil is degrading at an alarming rate (Zhu et al., 2019). In Northeast China, the plough layer thickness of black soil has decreased, while the soil organic matter (SOM) content has dropped (Tang et al., 2022). Some areas have even exhibited ‘broken skin yellowing’, as well as problems like soil erosion, soil hardening, and fertility decline (Jiang et al., 2020; Man et al., 2022; Zheng et al., 2018). This phenomenon was partly driven by traditional tillage, which exacerbated soil erosion through mechanical disturbance and the lack of sufficient organic materials supplementation. Meanwhile, traditional tillage caused severe soil disturbance that affected soil microbial activity and nutrient cycling (Jaskulska et al., 2023; Peng et al., 2023). To mitigate the negative effects of traditional tillage on the cultivated soil, sustainable agricultural practices have been implemented. Conservation tillage has been widely implemented as an effective practice in Northeast China (May et al., 2020).

Conservation tillage typically involves no tillage (NT) or reduced tillage techniques, while ensures that over 30% of the soil surface is covered with crop residues. This approach effectively minimizes soil erosion and conserves soil moisture (Lv et al., 2023). Compared with traditional tillage, conservation tillage enhances soil aeration, improves water retention, and promotes the formation of stable soil aggregates (Wang et al., 2020; Yuan et al., 2023). It also enhances soil nutrient levels and carbon accumulation (Dong et al., 2021; Han J. et al., 2024; Zhang K. et al., 2025). Additionally, conservation tillage fosters a favorable habitat for soil microorganisms by increasing microbial biomass and key enzyme activity, thereby optimizing the efficiency of nutrient cycling (Sekaran et al., 2020). In conclusion, conservation tillage provided important support for sustainable agriculture by regulating the soil environment in multiple dimensions. However, despite these benefits, challenges remain in colder regions. For example, full straw mulching hinder soil warming in early spring, which may negatively affect seed germination and early crop growth (Chen L. et al., 2021; Liu S. et al., 2022). To address these limitations, strip tillage (ST) has recently gained attention as a modified conservation tillage technique. ST reduces overall soil disturbance while retaining partial straw mulch, effectively mitigating early-season cold stress compared to conventional conservation tillage practices.

Strip tillage was used to conduct localized tillage only in the maize belt, while preserving straw mulching between rows to create an alternating distribution of maize belts and straw belts (Wang et al., 2023). Meanwhile, ST improved soil water–heat conditions, maintained soil structure (Chen Q. et al., 2021; Mikha et al., 2020), and further enhanced the environment for seed germination and growth (Cheng et al., 2024). In addition, soil organic carbon (SOC) content and microbial community structure under ST were closer to those under NT (Debska et al., 2020; Mackay et al., 2023), and there was a significant increase in soil microbial biomass and metabolic activity compared with traditional tillage (Balla Kovács et al., 2024). Furthermore, ST integrates traditional tillage and NT techniques, resulting in two distinct soil belts with different characteristics and functions (Pöhlitz et al., 2018). The maize belt creates favorable conditions for seed germination and plant growth, while the straw belt (Guo et al., 2010) serves to restore soil fertility, suppress weeds, reduce soil erosion, and increase microbial abundance (Han C. et al., 2024; Ranaivoson et al., 2017). However, current research on the impact of heterogeneous microenvironments formed by physical segmentation in ST on microbial community structure and function was still relatively lacking.

In the black soil region of Northeast China, seasonal freeze–thawing is a key abiotic stress factor driving the cyclical changes in soil physicochemical properties and biological processes (Liu M. et al., 2025; Sang et al., 2021). However, under traditional tillage practices, the lack of straw mulch exposes soil directly to the atmosphere. Consequently, although soil temperature rises rapidly in spring, significant soil moisture loss occurs, and the soil physical structure is prone to freeze–thawing damage. In contrast, the maize belts of ST warm up rapidly, facilitating crop germination, while the straw belts effectively retain soil moisture, maintain temperature, and mitigate freeze–thawing impacts (Chen et al., 2013). Therefore, this study investigates the effects of freeze–thawing stress on the structure, stability, and functional potential of soil microbial communities under the ST practice. Soil microorganisms play a central role in key biogeochemical cycling processes in terrestrial ecosystems, including critical ecological functions such as organic matter decomposition and nutrient cycling, and are important indicators for assessing soil health (Garcia Pichel, 2023). However, both tillage practices and freeze–thawing affect microbial community structure and function. Traditional conservation tillage increases soil microbial biomass carbon, microbial biomass nitrogen, and key enzyme activities of C, N and S cycles (Wen et al., 2024; Zhu et al., 2024), as well as enhances the abundance and diversity of soil microorganisms (Li et al., 2020). Recent studies have demonstrated that ST significantly increases the biomass of bacteria and fungi, especially that of arbuscular mycorrhizal fungi (Liu et al., 2024). Moreover, ST could better maintain the stability of microbial community compared to traditional tillage practice (Mackay et al., 2023). Furthermore, freeze–thawing affects soil structure by altering soil temperature and water phase change, and redistributes nutrients and mineral elements (Liu H. et al., 2025), or could cause physical damage to microorganisms (Klöffel et al., 2024). Meanwhile, freeze–thawing affected microbial biomass, community structure, and diversity (Ji et al., 2022; Min et al., 2023; Wu et al., 2020). However, there are relatively few studies on the changes in microbial community structure and function under the combined effect of freeze–thawing and ST.

Previously, relative quantitative amplicon sequencing was the main method in microbiomics research. However, with the development of technology, researchers developed absolute quantitative amplicon sequencing which could more comprehensively and precisely characterize microbial communities, thereby improving data accuracy (Maghini et al., 2024). In this study, we used Accu16S™ bacteria and AccuITS™ fungi absolute quantitative sequencing to clarify the effects of ST (straw belts and maize belts) on soil microbial community structure and functioning under freeze–thawing drive. Therefore, we proposed the following hypotheses: (1) Freeze-thawing can regulate the differences in microbial community structure and function induced by ST; (2) ST creates a synergistic system of “low disturbance-straw mulching,” forming a microenvironment that buffers freeze–thawing and thereby further enriching microbial taxa with nitrogen and sulfur cycling functions.

## 2 Materials and methods

### 2.1 Experimental location

This research was based on a field experimental conducted in 2018, in San-Ke-Shu Village, Lishu County, Jilin Province, China (43°2′N, 123°5′E). During the 2018–2021 maize growing season (late April to early October), the average temperature was 18.3 °C with an average annual rainfall of 513 mm in this study region. The soil was classified as meadow black soil according to the United States Soil Taxonomy (Ditzler, 2017). Initial soil physicochemical properties have been reported in a previous study (Sha et al., 2024). In the 0–20 cm soil layer, the texture was characterized by high clay content (45.6%), high silt content (41.6%), and low sand content (12.8%). Baseline soil properties before the experiment were: pH 5.9 (1:2.5 soil-to-water ratio), organic carbon 27.3 g kg^−1^, total nitrogen 1.4 g kg^−1^, Olsen-P 36.9 mg kg^−1^, and available potassium 159 mg kg^−1^.

### 2.2 Experimental design

In this study, two tillage practices—rotary tillage (RT) and strip tillage (ST)—were evaluated using a completely randomized block design with three replicates. RT had no straw mulch and ST had complete straw mulch to the field. Each plot covered an area of 96 m^2^ (length 20 m × width 4.8 m) and 8 rows of corn crops, with a spacing of 0.6 m between each two rows. Maize (*Zea mays* L. cv. De-Mei-Ya 3) was sown with a row spacing of 0.6 m at a planting density was controlled of 70,000 ha^−1^. Fertilization and other field management practices followed standard protocols and are described in detail in a previous study (Sha et al., 2024).

### 2.3 Soil sampling and measurements

**Figure 1:**
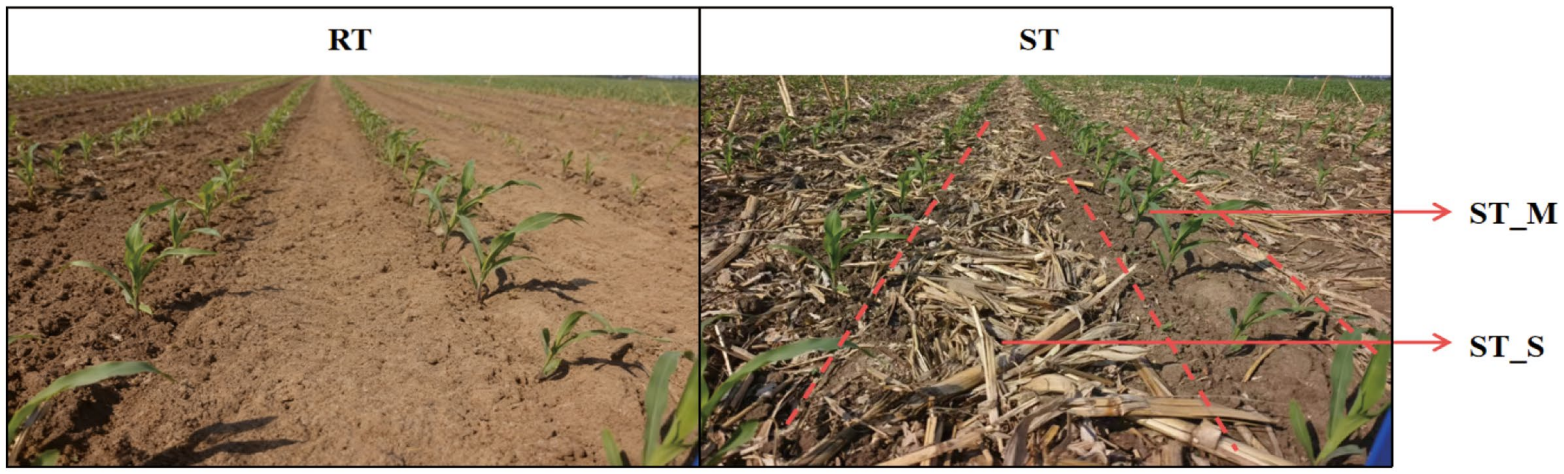
Schematic diagram of different tillage practices. RT, Rotary tillage; ST-M, Maize belt of strip tillage; ST-S, Straw belt of strip tillage; ST, Strip tillage.

Soil samples from the 0–20 cm layer were collected in October 2020 (before freeze–thawing) and April 2021 (after freeze–thawing) from four treatment areas: RT, the maize belt of strip tillage (ST-M, located between adjacent maize rows), the straw belt of strip tillage (ST-S, located between adjacent crop rows), and ST (evenly combined from both maize and straw belts; Figure 1). The ambient temperature during the sampling periods ranged from approximately −4 °C to 7 °C in October 2020 and from −5 °C to 5 °C in April 2021. Within each plot, five sampling points were selected in an “S”-shaped pattern to ensure representativeness. These two sampling periods were taken before and after the freeze–thawing, and the study site was situated within a mid-to-high latitude region where obvious freeze–thawing process were consistently observed during winter periods. The collected soil samples were divided into three portions. The first was stored at −80 °C for subsequent high-throughput sequencing. The second was kept at 4 °C for mineral Nitrogen (MN) determination; and the remaining portion was reserved for analyzing other physicochemical properties. These samples were firstly air-dried at room temperature passed through a 2-mm sieve for measurement of pH, electric conductivity (EC), available phosphorus (AP), and available potassium (AK), and then finely ground to pass through a 0.15-mm sieve for the determination of SOC, total nitrogen (TN), total phosphorus (TP), and KMnO₄-oxidizable C (ROC). Specifically, soil pH and EC were determined a in 1:5 soil-to-water suspension after shaking at 25 °C for 3 min. AP was measured by colorimetric analysis following extraction of soil with 0.5 mol L^−1^ NaHCO_3_, while AK was extracted using 1.0 mol L^−1^ CH_3_COONH_4_ and subsequently quantified (Tian et al., 2021). The assessments of SOC, TN, TP and MN were conducted according to the methods described by Luo et al. (2018). All soil samples from different sampling periods were treated as independent samples, and subsequent analyses were conducted independently following the grouping of “sampling period + tillage treatment” (Lauber et al., 2010).

### 2.4 DNA extraction, high-throughput amplicon

The specific steps were as follows: Accu16S™ (Accurate 16S absolute quantification sequencing) and AccuITS™ (Accurate ITS absolute quantification sequencing) were performed by Genesky Biotechnologies Inc., Shanghai, 201315 (China). Total genomic DNA was extracted using the FastDNA™ SPIN Kit for Soil (MP Biomedicals, Santa Ana, CA). The integrity of genomic DNA was assessed through agarose gel electrophoresis while the concentration and purity of genomic DNA were detected through the Nanodrop 2000 and Qubit3.0 Spectrophotometer. Also, an appropriate proportion of spike-ins mixture with known gradient copy numbers were added to the sample DNA. The V3-V4 hypervariable regions of the 16S rRNA gene and spike-ins were amplified with the primers 341F (5′-CCTACGGGNGGCWGCAG-3′) and 805R (5′-GACTACHVGGGTATCTAATCC-3′) and the ITS2 hypervariable regions of the fungal community and spike-ins were amplified with the primers ITS2 (5′-GCATCGATGAAGAACGCAGC-3′) and ITS2 (5′-TCCTCCGCTTATTGATATGC-3′) and then sequenced using Illumina NovaSeq 6000 sequencer. The PCR programmer was as follows: denaturation at 94 °Cor 2 min; 25 cycles of 94 °C for 30 s, 55 °Cfor 30 s, elongation at 72 °C for 1 min; and a final extension at 72 °C for 10 min. The PCR products were identified using 2% agarose gel electrophoresis. Purification was done using the AxyPrep DNA Gel Extraction Kit (Axygen Biosciences, Union City, CA, United States) and quantified using QuantiFluor™-ST (Promega, United States) following the manufacturer’s instructions. The PCR products were purified before being used to build an amplicon library in an equimolar concentration. Subsequently, paired-end sequencing (2 × 250 bp) was performed using the Illumina MiSeq sequencing platform. The raw read sequences were processed in QIIME2 (Bolyen et al., 2019) while the adaptor and primer sequences were trimmed using the cutadapt plugin, statistics on the quality of raw data. DADA2 (Callahan et al., 2016) plugin was used for quality control and to identify amplicon sequence variants (ASVs). Taxonomic assignments of bacterial ASV representative sequences were performed with a confidence threshold of 0.8 using a pretrained Naive Bayes Classifier, while those of fungal ASV representative sequences were performed with a confidence threshold of 0.6 using the same classifier. This classifier was selected due to its efficiency and accuracy in adapting to cross-taxon high-throughput data, and it is fully scientifically valid: the classifier is pre-trained on specialized databases for the two microbial groups, respectively, (RDP for bacteria and UNITE for fungi), with the highest achievable taxonomic level being the species level. Then, the spike-in sequences were identified and reads were counted. Standard curves for each sample were generated based on the read counts vs. spike-in copy number, and the absolute copy number of each ASV in each sample was calculated by using the read counts of the corresponding ASV. Since the spike-in sequence is not a component of the sample flora, it was removed from subsequent analysis (Jiang et al., 2019). Furthermore, the “absolute abundance” in this study refers to the quantified absolute copy number of specific functional traits (or corresponding microbial taxa) per unit sample (e.g., per gram of soil). It is calculated based on the Accu16S/AccuITS absolute quantification sequencing technology, reflecting the actual quantity of functional microorganisms in the environment rather than their relative proportional relationships.

### 2.5 Statistical analysis

To compare the *α*-diversity of soil microbial communities, the Shannon diversity index was calculated using the R package ‘vegan’. To assess soil bacterial and fungal *β*-diversities, non-metric multidimensional scaling (NMDS) was used for visualization, while analysis of similarities (ANOSIM) and permutational multivariate analysis of variance (PERMANOVA, also known as ADONIS) based on Bray-Curtis dissimilates were applied to quantitatively evaluate community differences among groups. To compare differential microbial abundance and identify potential biomarkers, Linear Discriminant Analysis Effect Size (LEfSe) analysis was performed via the Galaxy we interface,^1^ using a significance threshold of *p* < 0.05 and an LDA score > 2. Additionally, a Mantel test was conducted using the ‘ggcor’ package of the R software, employing the mantel_test function for statistical analysis and visualization. FAPROTAX was used to predict ecologically relevant functions of bacterial and archaeal taxa based on 16S rRNA gene amplicon sequencing data (Louca et al., 2016). For Fungal communities, FungalTraits, a functional database that supports assignment at both the genus and species hypothesis level, was used to facilitate rapid ecological functional annotation in environmental studies (Põlme et al., 2020). The means and standard deviations of the data were calculated and statistically analyzed using one-way analysis of variance (ANOVA) and Tukey’s multiple comparison analysis using IBM SPSS 20.0 (IBM Corporation, SA). The significance level was set at *p* < 0.05, unless otherwise stated.

## 3 Results

### 3.1 Soil microbial community diversity

**Figure 2:**
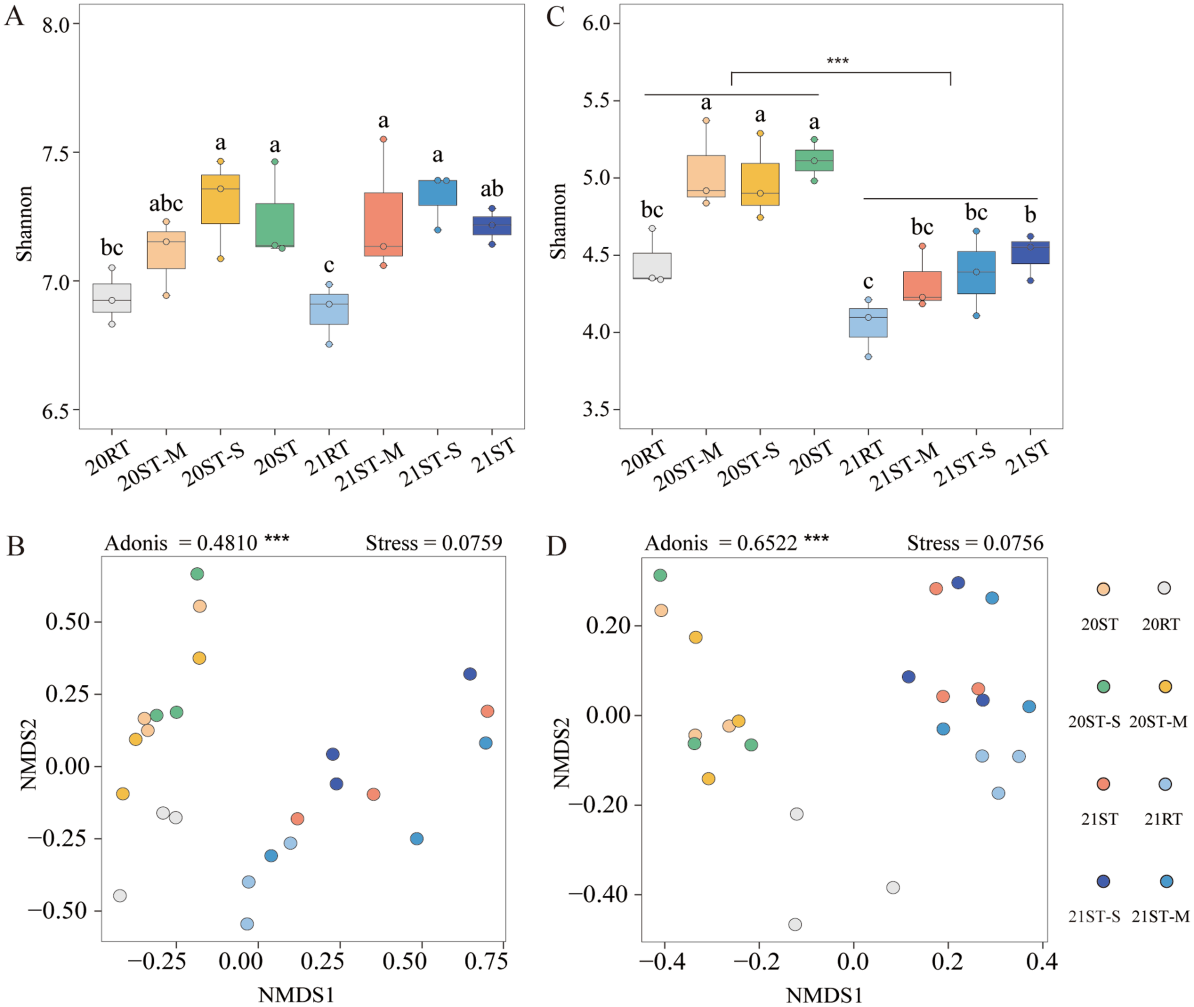
Shannon index of *α*-diversity and non-metric multidimensional scaling (NMDS) index of *β*-diversity of the bacterial **(A,B)** and fungal **(C,D)** communities in 2020 and 2021. Various lowercase letters indicate significant differences among different treatments (*p* < 0.05) and different asterisks indicate significant differences at *p < 0.05, **p < 0.01, and ***p < 0.001. 2020, 20; 2021, 21; RT, rotary tillage; ST-M, maize belt of strip tillage; ST-S, straw belt of strip tillage; ST, strip tillage.

In 2020, the ST treatment significantly increased the Shannon index of bacterial and fungal communities compared to the RT treatment (*p* < 0.05), while no significant difference was observed between the ST-M and ST-S treatments (Figure 2). However, the difference in the Shannon index of fungal communities between the RT and ST treatments gradually decreased in 2021 (Figure 2C), but remained statistically significant (*p* < 0.05). Furthermore, freeze–thawing had a more pronounced impact on the diversity of soil fungal communities than on bacterial communities (*p* < 0.001). NMDS was used to analyze the effects of tillage practices and sampling periods on soil bacterial and fungal community structures. The results showed significant differences in the microbial community structure between 2020 and 2021. The Adonis values in Figure 2B (bacterial) and 2D (fungal) were 0.4810 and 0.6522, respectively (*p* < 0.001), and the stress values were both below 0.2, indicating the reliability of the ordination results. Meanwhile, microbial groups corresponding to different tillage practices and sampling periods showed clear clustering and separation in the NMDS space. These results suggest that freeze–thawing narrowed the difference in fungal community structure between the RT and ST treatments compared to the bacterial community.

### 3.2 Soil microbial community composition

**Figure 3:**
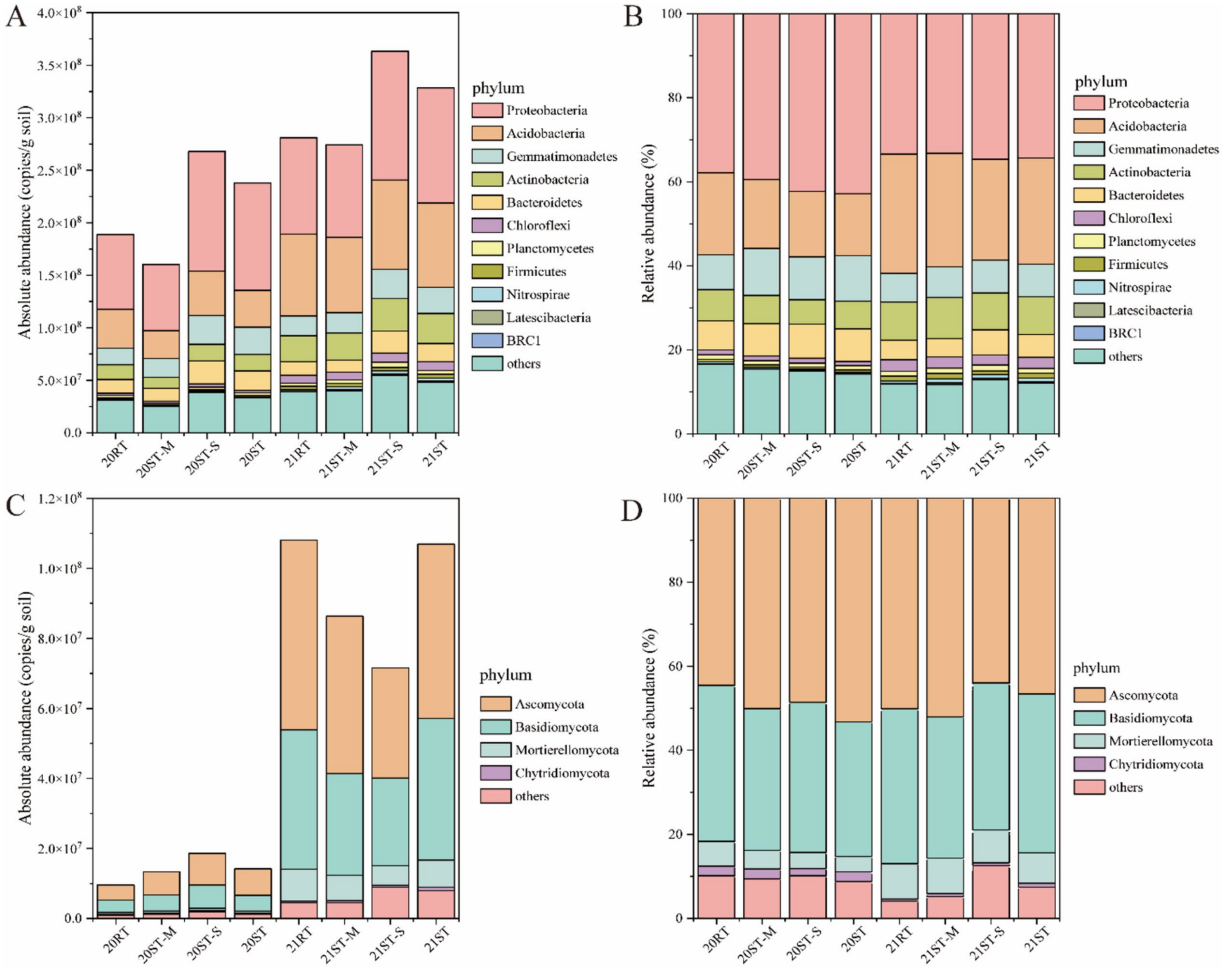
The absolute abundance and relative abundance of dominant phyla in bacterial and fungal communities under different tillage practices in 2020 and 2021 **(A)** Absolute abundance of bacteria. **(B)** Relative abundance of bacteria. **(C)** Absolute abundance of fungi. **(D)** Relative abundance of fungi. 2020, 20; 2021, 21; RT, rotary tillage; ST-M, maize belt of strip tillage; ST-S, straw belt of strip tillage; ST, strip tillage.

This study selected phyla that showed differences among treatments for presentation. The estimated absolute copy numbers per gram of soil are shown in Figures 3A,C, while the corresponding relative abundances (RAs) at phylum level are shown in Figures 3B,D. The main bacterial phyla included Proteobacteria (32.12–42.95%), Acidobacteria (14.92–27.74%), Gemmatimonadetes (6.72–11.16%), Actinobacteria (5.89–9.48%), Bacteroidetes (4.21–8.18%), Chloroflexi (1.01–2.71%), Planctomycetes (0.97–1.41%), Firmicutes (0.50–1.22%), Nitrospirae (0.28–0.99%), Latescibacteria, and BRC1 (Figures 3A,B). Meanwhile, the main fungal phyla included Ascomycota (43.99–53.23%), Basidiomycota (32.03–37.84%), Mortierellomycota (3.64–8.48%), and Chytridiomycota (0.46–2.37%; Figures 3C,D). Compared with the RT treatment, the ST treatment significantly decreased the RAs of Acidobacteria and Nitrospirae (*p* < 0.05), while significantly increasing the relative abundance (RA) of Gemmatimonadetes (*p* < 0.05) in 2020. Meanwhile, the absolute abundance (AA) of BRC1 in the ST-S treatment was higher than that in the ST-M treatment (*p* < 0.05). In 2021, compared with the RT treatment, the ST treatment significantly decreased the RA of Acidobacteria, and significantly increased the absolute abundances (AAs) of Nitrospirae and Latescibacteria (*p* < 0.05). Meanwhile, the ST-S treatment significantly increased the RA of Bacteroidetes compared with the ST-M treatment (*p* < 0.05). In addition, compared with the RT treatment, the ST, ST-M and ST-S treatments also significantly increased the AAs of Nitrospirae and Latescibacteria (*p* < 0.05). Compared to 2020, in 2021, the AA and RA of Chloroflexi significantly increased in the RT, ST-M, ST-S, and ST treatments, while those of Nitrospirae significantly increased in the ST-M, ST-S, and ST treatments (*p* < 0.05). Additionally, the AAs of Firmicutes, Ascomycota, and Mortierellomycota significantly increased in the RT, ST-M, ST-S, and ST treatments; the AA of Latescibacteria was also significantly increased in the ST-M, ST-S, and ST treatments. Furthermore, the RA of Acidobacteria significantly increased, whereas the RA of Proteobacteria significantly decreased in the RT, ST-M, ST-S, and ST treatments (*p* < 0.05).

**Figure 4:**
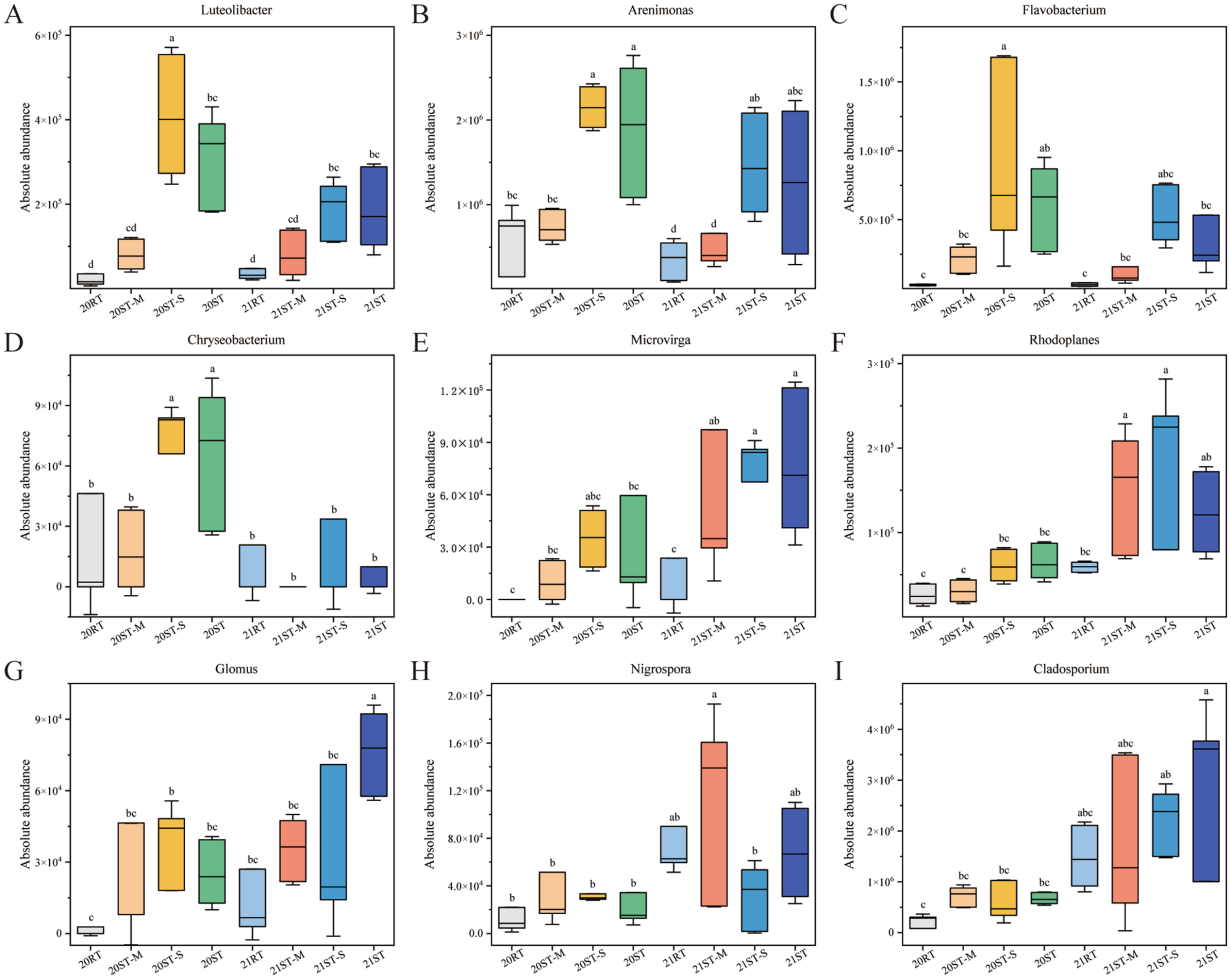
Analysis of species differences of bacteria **(A–F)** and fungi **(G–I)** under different tillage practices in 2020 and 2021. Various lowercase letters indicate significant differences among different treatments (*p* < 0.05). 2020, 20; 2021, 21; RT, rotary tillage; ST-M, maize belt of strip tillage; ST-S, straw belt of strip tillage; ST, strip tillage.

ANOVA was employed to compare the taxa of different treatments at the genus level, and to identify genera that exhibited significant differences among treatments (Figure 4). In 2020, compared with the RT treatment, the ST treatment significantly increased the AAs of *Luteolibacter*, *Arenimonas*, *Flavobacterium*, and *Chryseobacterium* (*p* < 0.05). The ST-S treatment significantly increased the AAs of *Luteolibacter*, *Arenimonas*, *Flavobacterium*, and *Chryseobacterium* compared to the ST-M treatment (*p* < 0.05). In 2021, the ST treatment significantly increased the AAs of *Luteolibacter*, *Arenimonas*, *Microvirga*, and *Glomus* compared to the RT treatment (*p* < 0.05). The ST-S treatment significantly increased the AAs of *Arenimonas* compared to the ST-M treatment; conversely, the ST-M treatment significantly increased the AAs of *Nigrospora* compared to the ST-S treatment (*p* < 0.05). Among them, the ST and ST-S treatments significantly increased the AAs of *Flavobacterium* and *Chryseobacterium* compared to the RT treatment in 2020, however, this trend became insignificant in 2021 (*p* < 0.05). Compared with the RT treatment, the ST and ST-S treatments significantly increased the AA of *Luteolibacter* and *Arenimonas*, and this trend kept consistent in 2020 and 2021 (*p* < 0.05). In 2020, the ST treatment increased the AAs of *Microvirga* and *Glomus* compared to the RT treatment, and this increased trend became significant in 2021 (*p* < 0.05). The AA of two fungi, *Nigrospora* and *Cladosporium* also increased after freeze–thawing.

### 3.3 Soil environmental factors influencing microbial community

There are various differences in soil chemical properties between the two tillage practices of the RT and ST treatments, as well as between their ST-M and ST-S treatments (Table 1). In 2020, compared with the RT treatment, the ST treatment significantly increased soil EC, SOC, TP, ROC, and AK; the ST-S treatment significantly increased ROC and AK (*p* < 0.05). Data collected in 2021 showed that the ST treatment significantly higher SOC than the RT treatment, while the ST and ST-S treatments had significantly higher TN than the RT and ST-M treatments (*p* < 0.05). Furthermore, the ST-M treatment had a higher MN content, whereas the ST-S treatment maintained higher TN and pH levels (*p* < 0.05). This phenomenon reflects the impact of straw distribution. Notably, compared with 2020, most soil nutrient indicators showed a decreasing trend in 2021, while SOC and TN exhibited an overall increasing trend.

**Table 1:** Soil chemical properties under different tillage practices.

Factors	2020				2021			
	RT	ST-M	ST-S	ST	RT	ST-M	ST-S	ST
pH	6.39 a	6.12 bc	6.26 ab	6.18 ab	5.91 cde	5.74 e	6.04 bcd	5.89 de
EC (μs/cm)	80.07 c	143.00 a	120.43 b	133.63 ab	45.40 d	43.57 d	48.37 d	45.83 d
SOC (g/kg)	12.87 c	13.70 bc	14.19 bc	14.44 b	14.87 b	16.67 a	16.78 a	17.10 a
TN (g/kg)	1.02 c	1.09 c	1.09 c	1.12 bc	1.06 c	1.10 c	1.21 b	1.35 a
TP (g/kg)	0.75 bc	0.82 b	0.81 b	0.91 a	0.72 c	0.78 bc	0.80 b	0.78 bc
ROC (mg/g)	3.00 c	3.74 abc	4.39 ab	4.51 a	3.31 c	3.23 c	3.56 bc	3.73 abc
MN (mg/kg)	29.54 ab	23.93 abc	31.37 a	27.43 abc	26.13 abc	23.88 abc	20.38 c	21.71 bc
AP (mg/kg)	60.93 a	49.43 ab	59.06 a	60.73 a	30.94 c	39.28 bc	35.35 bc	43.21 abc
AK (mg/kg)	185.67 bc	228.67 ab	269.00 a	253.33 a	134.67 cd	105.33 d	116.33 d	120.00 d

EC, Electric conductivity; SOC, soil organic carbon; TN, total nitrogen; TP, total phosphorus; ROC, KMnO₄-oxidizable C; MN, mineral nitrogen; AP, available phosphorus; AK, available potassium. Various lowercase letters indicate significant differences among different treatments (*p* < 0.05). RT, rotary tillage; ST-M, maize belt of strip tillage; ST-S, straw belt of strip tillage; ST: strip tillage.

**Figure 5:**
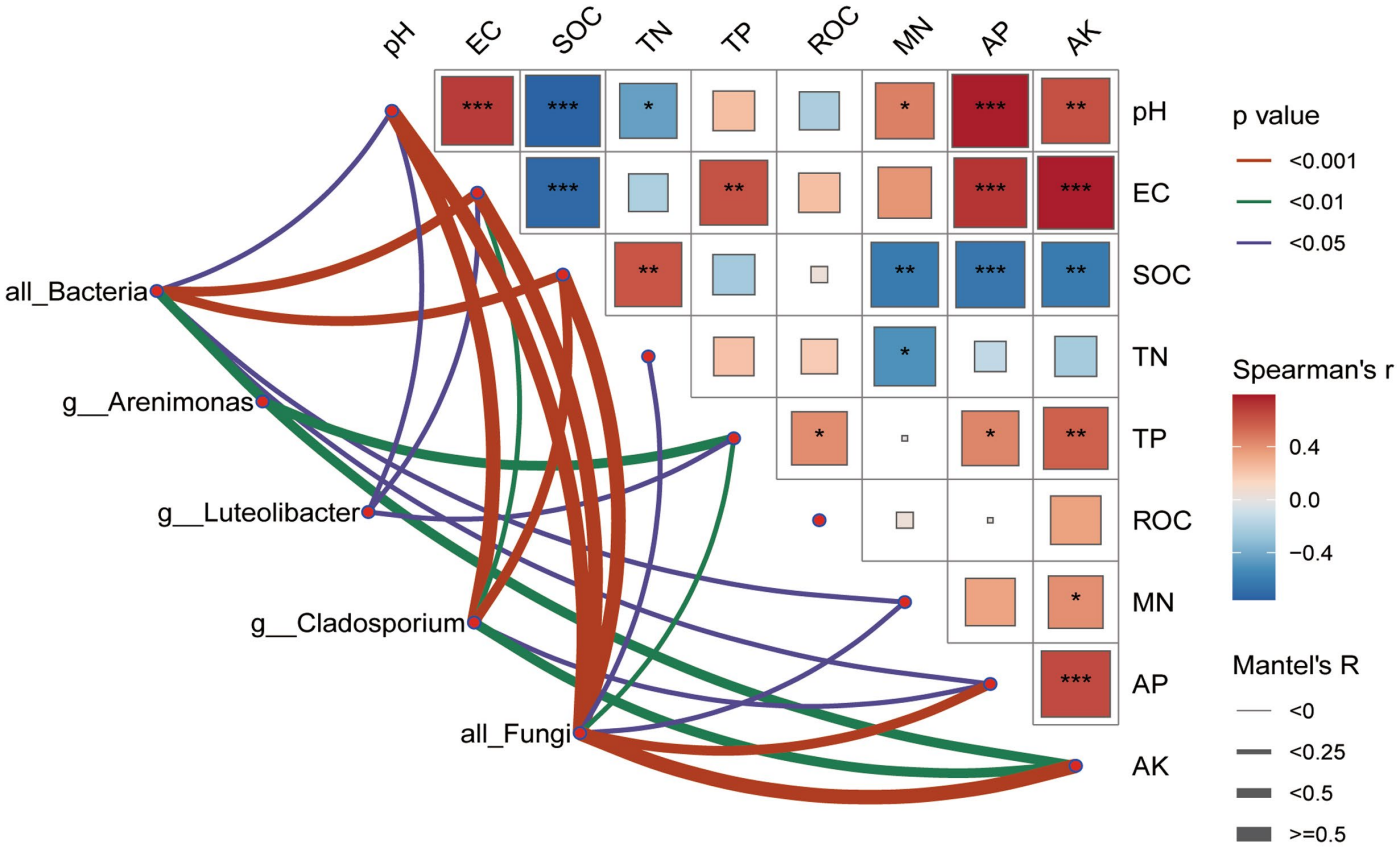
Mantel tests revealing correlations between microbial community structure and soil physicochemical properties. EC, electric conductivity; SOC, soil organic carbon; TN, total nitrogen; TP, total phosphorus; ROC, KMnO_4_-oxidizable; MN, mineral nitrogen; AP, available phosphorus; AK, available potassium. Different asterisks indicate significant differences at *p < 0.05, **p < 0.01, and ***p < 0.001.

This study employed the Mantel test to explore the correlations between microbial community composition, indicator groups, and environmental factors under different treatments, thereby identifying the key environmental drivers influencing changes in community structure (Figure 5). The soil bacterial community showed an extremely significant correlation with EC and SOC (*p* < 0.001), a highly significant correlations with AK (*p* < 0.01), and significant correlations with pH, MN, and AP (*p* < 0.05). By contrast, the soil fungal community exhibited extremely significant associations with pH, EC, SOC, AK, and AP (*p* < 0.001), highly significant associations with TP (*p* < 0.01), and significant associations with TN (*p* < 0.05). At the genus level, *Arenimonas* was highly significantly correlated with TP (*p* < 0.01); *Luteolibacter* was significantly correlated with pH, EC and TP (*p* < 0.05); and *Cladosporium* was extremely significantly correlated with pH and SOC, highly significantly correlated with EC and AK (*p* < 0.01), and significantly correlated with AP (*p* < 0.05).

### 3.4 Microbial community biomarkers

**Figure 6:**
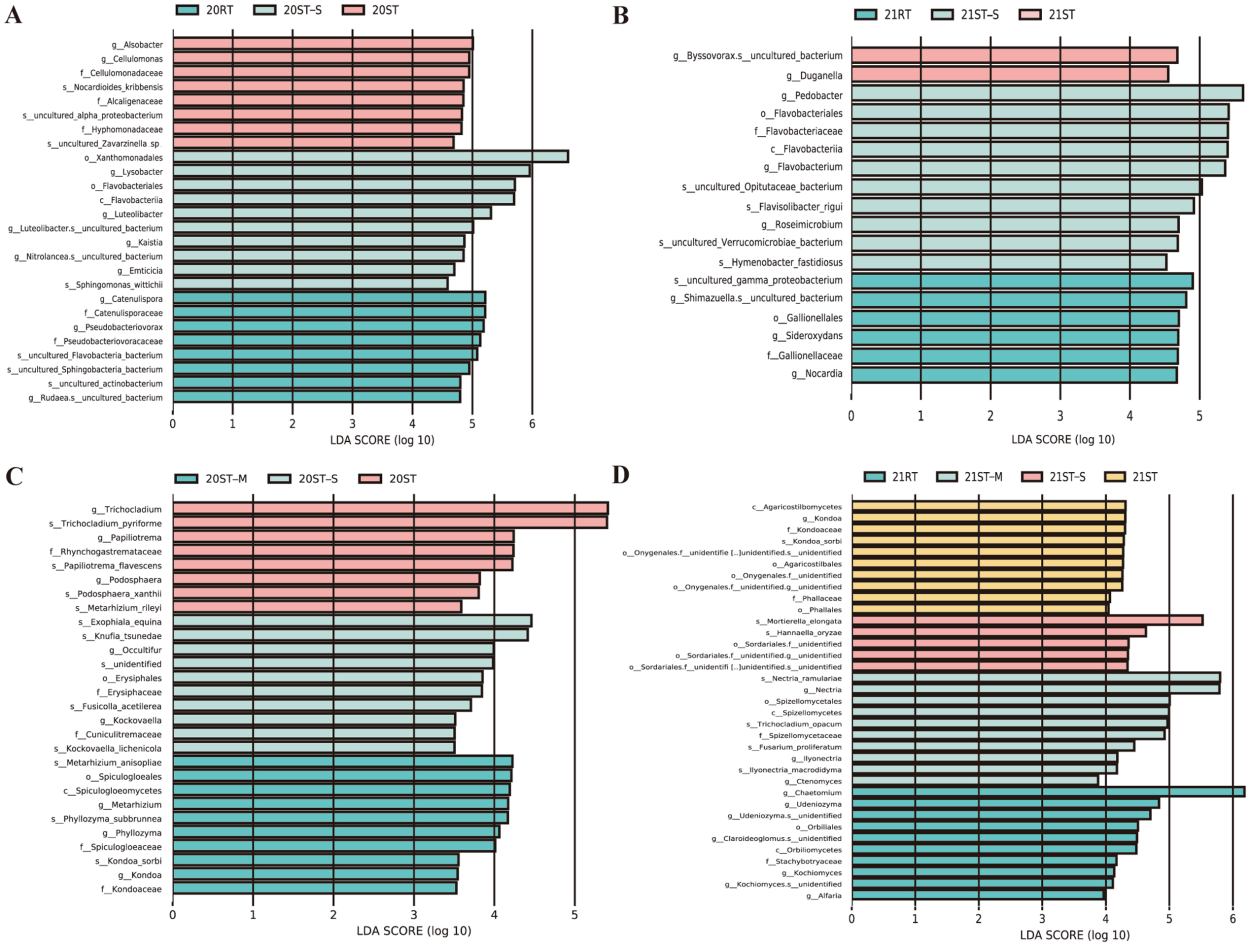
Linear discriminant analysis (LDA) of bacterial **(A,B)** and fungal **(C,D)** communities under different tillage practices in 2020 and 2021, determined with a threshold value of |LDA| > 3 and *p* < 0.05. 2020, 20; 2021, 21; RT, rotary tillage; ST-M, maize belt of strip tillage; ST-S, straw belt of strip tillage; ST, strip tillage.

LEfSe was used to analyze the biomarker microbial at the genus level among the treatments, with the threshold set as |LDA| > 3 and *p* < 0.05 (Figure 6). There was significant enrichment of *Catenulispora*, *Pseudobacteriovorax*, and *Rudaea* in the 20RT treatment; *Trichocladium*, *Papiliotrema*, and *Podosphaera* in the 20st-M treatment; *Lysobacter*, *Luteolibacter*, *Kaistia*, *Nitrolancea*, *Emticicia Occultifur*, and *Kockovaella* in the 20st-S treatment; *Alsobacter*, *Cellulomonas*, *Metarhizium*, *Phyllozyma*, and *Kondoa* in the 20st treatment; *Byssovorax*, *Duganella*, *Chaetomium*, *Udeniozymag*, *Claroideoglomus*, *Kochiomyces*, and *Alfaria* in the 21RT treatment; *Nectriao*, *llyonectria*, and *Ctenomyces* in the 21st-M treatment; *Pedobacter*, *Flavobacterium*, and *Roseimicrobium* in the 21st-S treatment; *Shimazuella*, *Sideroxydans*, *Nocardia*, and *Kondoa* in the 21st treatment.

### 3.5 Microbial community functional prediction

**Figure 7:**
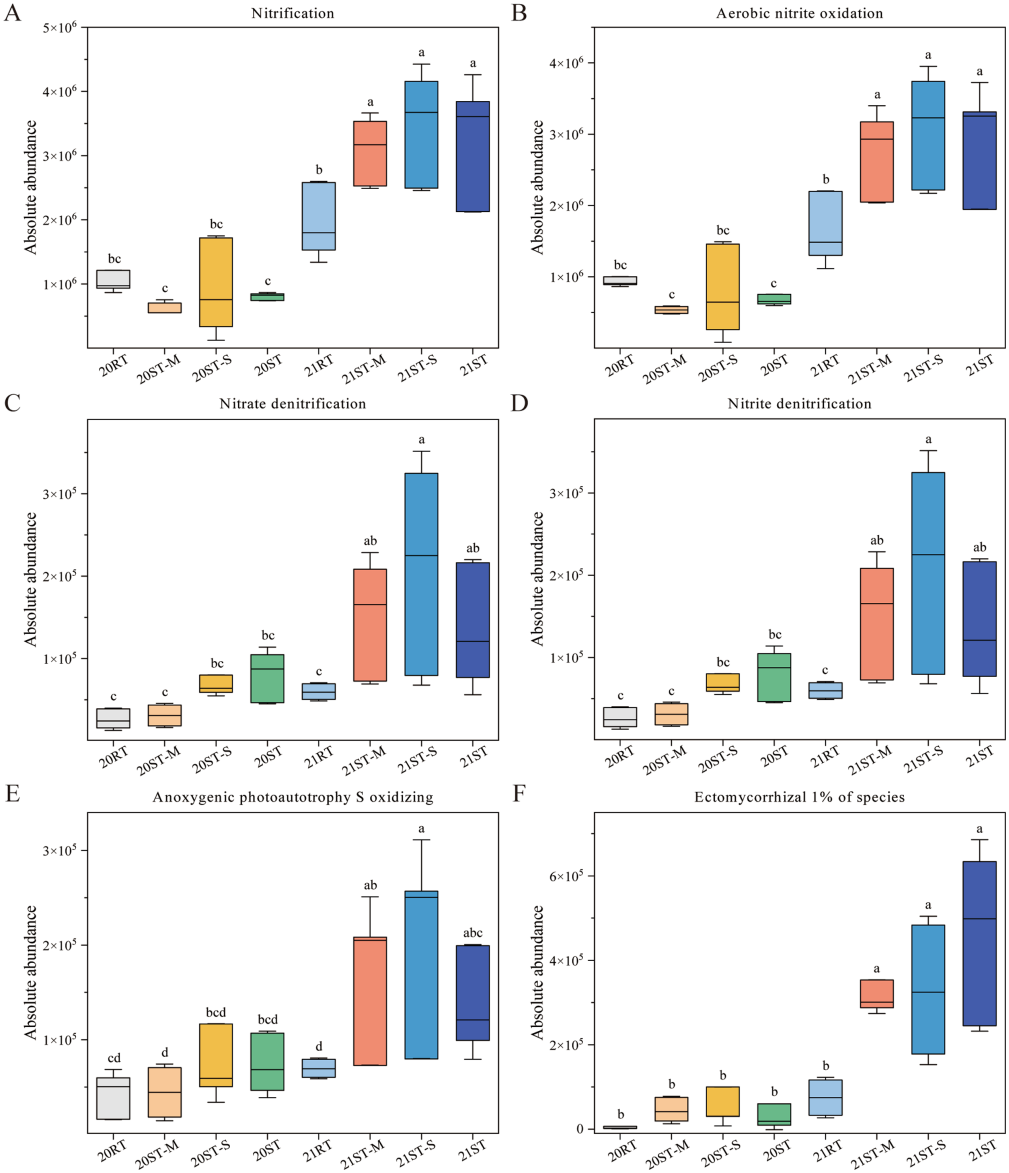
Prediction of microbial functions under different tillage practices. **(A–E)** Prediction of bacterial FAPROTAX functions; **(F)** prediction of fungal FungalTraits functions. Various lowercase letters represent significant differences among all treatments in different treatment (*p* < 0.05). 2020, 20; 2021, 21; RT, rotary tillage; ST-M, maize belt of strip tillage; ST-S, straw belt of strip tillage; ST, strip tillage.

According to the FAPROTAX functional predictions, the ST-M, ST-S, and ST treatments significantly increased the AAs of the nitrification, aerobic nitrite oxidation, nitrate denitrification, nitrite denitrification, and anoxygenic photoautotrophy S oxidizing functions compared to the RT treatment in 2021 (Figures 7A–E; *p* < 0.05). This indicated that the application of straw mulching may have increased the abundance of this particular functional bacterial community. The fungal functional prediction analysis revealed that the ST-M, ST-S, and ST treatments significantly increased the AA of ectomycorrhizal function compared to the RT treatment, although it constituted a mere 1% of the microbial community (Figure 7F; *p* < 0.05).

## 4 Discussion

### 4.1 Effects of strip tillage on soil microbial community diversity

The diversity index is an important indicator for characterizing the soil microbial communities, and its variations is jointly regulated by historical and current factors (Sun et al., 2014). In our study, the ST and ST-S treatments significantly increased the *α*-diversity index of microbial communities in 2020 (Figures 2A,C). These results above were similar to previous studies, which found an increase in microbial α-diversity under conservation tillage practices (Li T. et al., 2021a; Sommermann et al., 2018). This phenomenon might be attributed to ST reducing soil disturbance, thereby providing an optimal environment for soil microbial colonization (Ghimire et al., 2023). This study site is located in a typical mid-to-high latitude region where the study soil experience pronounced seasonal freeze–thawing due to prolonged winter. These seasonal dynamics can result to significant shifts in microbial community structure among sampling periods. Compared to 2020, the differences in the *α*-diversity of fungal community among treatments showed a decreased in 2021 (Figure 2C). This observation is consistent with previous studies that have reported increased fungal abundance during winter (Deng et al., 2024). One possible explanation is that freeze–thawing induce changes in soil water phases, thereby altering microbial community composition (Ji et al., 2022; Ren et al., 2020). Specifically, soil freeze–thawing disrupts soil aggregate structure, releases protected organic matter, and simultaneously alters soil pore connectivity and water phase state, thereby promoting the contact between straw and microorganisms and directly affecting the rate and efficiency of straw decomposition (Dong C. J. et al., 2023; Zhang Y. et al., 2024). Meanwhile, the nutrients released during straw decomposition further regulate the structure and function of microbial communities (Zhang W et al., 2025). Overall, these findings demonstrate that freeze–thawing is a key factor influencing soil fungal α-diversity.

In this study, microbial community structure was influenced by both sampling periods and tillage practices (Figures 2B,D). Previous research has shown clear differences in microbial community composition between conservation tillage and traditional tillage (Liu Z et al., 2022), and the variations observed in this study support those findings. These differences may be attributed to the impact of tillage practices on the soils physical structure, which in turn affects microbial habitat and distribution (Oliveira et al., 2024; Wen et al., 2024). Meanwhile, significant differences were observed in microbial community structure across sampling periods, with these variations being regulated by the freeze-thaw process. Specifically, during freeze-thaw cycles, permafrost acts as a physical barrier, limiting gas exchange between the soil microbial communities and the atmosphere, and reducing the availability of water to microbes. These environmental constraints can lead to a decline in microbial biomass and metabolic activity (Min et al., 2023; Wang et al., 2019). Additionally, freeze-thaw also influences the structure of the soil microbial community by affecting soil temperature (Klöffel et al., 2024). The above results indicate that the stable and higher microbial α-diversity under ST implies stronger functional redundancy, enabling the soil ecosystem to maintain core nutrient cycling functions when encountering environmental disturbances. Meanwhile, the significant changes in microbial *β*-diversity induced by freeze-thawing may enhance the spatial heterogeneity of microbial communities, thereby providing a buffer mechanism for the ecosystem to respond to environmental changes. In brief, this study showed that freeze-thawing narrowed differences in fungal community diversity between RT and ST treatments. This result suggests that seasonal temperature fluctuations may weaken the regulatory effect of tillage practices on the microbial community, thereby affecting the functional stability of the ecosystem.

### 4.2 Effects of strip tillage on soil microbial community composition

The results of this study showed that the ST treatment significantly reduced the RA of Acidobacteria (Figure 3B), which contrasts with previous findings on conservation tillage (Li M. et al., 2021). Most Acidobacteria are oligotrophic microorganisms, typically more competitive in nutrient-poor soils or prefer degrading recalcitrant SOM (Wang et al., 2017). This suggests that the decomposition of returned straw creates a nutrient-rich environment, leading to a decline in the abundance of Acidobacteria under ST. However, compared with 2020, the RA of Acidobacteria significantly increased in 2021, while that of Proteobacteria significantly decreased. This shift can be attributed to the reduction in available soil nutrients during winter, which gradually transformed the soil microenvironment into an oligotrophic state. This study also confirms this result (Table 1). Moreover, the ST treatment significantly increased the RA of Gemmatimonadetes and the AA of Latescibacteria and Chytridiomycota. A previous study demonstrated that Gemmatimonadetes exhibited a higher abundance in NT soil (Essel et al., 2018) and showed a positive correlation with crop residues. This may be due to their possession of multiple cellulose, hemicellulose and polysaccharide degradation genes, which played a role in the initial degradation and hydrolysis of complex organic carbon compounds (Baker et al., 2015). Meanwhile, Latescibacteria demonstrated significant capabilities for degrading proteins, lipids, and polysaccharides (Farag et al., 2017). It was regarded as a potential indicator of soil fertility and quality when straw was returned to the soil (Yu et al., 2018). This finding was also observed in our study, where the ST significantly increased the AA of Latescibacteria in 2021. Furthermore, the ST treatment significantly increased the AA of Chytridiomycota (Figure 3A), which is associated with its ability to degrade cellulose (Kameshwar et al., 2016). These phenomena may be related to the increased content of recalcitrant organic compounds, such as aromatic compounds and lignin under straw return (Xu et al., 2020), and are regulated by soil moisture conditions. Previous studies have shown that moisture is a key limiting factor influencing microbial survival, metabolism, and soil nutrient release during the freeze-thawing process (Chang et al., 2022; Liu et al., 2024). Within the ST system, the ST-S treatment significantly increased the AA of BRC1 and the RA of Proteobacteria and Bacteroidetes compared to the ST-M treatment. This might be attributed to the accumulation of straw in the ST-S treatment, which provided suitable substrate resources and created a favorable microenvironment for the survival of these microbial taxa (Khan et al., 2023). Previous studies demonstrated that BRC1 can utilize recalcitrant organic matter as a carbon source to cope with environmental stress, and is more adaptable to extreme habitats (Chávez-Romero et al., 2016). Proteobacteria are part of the copiotroph bacteria (r-strategists) that could convert residues into simpler compounds during the initial stages of decomposition (Ahn et al., 2016). Previous research results have shown that the RA of Bacteroidetes tends to usually increased with the release of organic matter from crop roots (Nguyen et al., 2016), and this bacterial group is also capable of degrading cellulose (Li T. et al., 2021b). Furthermore, compared to 2020, the ST significantly increased the RA of Nitrospirae in 2021. This phenomenon may be attributed to the fact that freeze-thawing alter the physical properties of the soil, creating a more favorable microhabitat for Nitrospirae. Meanwhile, ST optimizes the uniformity of freeze-thawing, promotes sufficient contact between straw-soil, and facilitates the release of abundant nutrients from straw, thereby further increasing the RA of this phylum. Addition, freeze-thaw cycles significantly increased the AA of fungal communities (Figure 3D), which may be associated with reduced physical disturbance of fungal mycelia networks under NT practices (Li Y. et al., 2021). In summary, the variations in the microbial community abundance between treatments reflect the impact of tillage practices and freeze-thawing on soil microbial communities to some extent.

In cold regions with low temperatures, the focus of straw-degrading microorganisms should be on cold-tolerant or cold-loving microorganisms, such as the dominant *Luteolibacter*, and *Arenimonas*. These genera can participate in the N cycle and might maintained microbial community stability by reducing the nitrate toxicity (Xu et al., 2023), and decompose organic matter to release nutrients (e.g., N and P) into the soil (Zadel et al., 2020), thereby contributing to the improvement of soil physical properties (Figures 4, 5). Notably, these two bacterial genera exhibit strong resistance to freeze-thawing and could serve as indicator taxa for ST. Under the ST system (the ST and ST-S treatments), straw mulching provides abundant organic substrates and nutrients, laying the foundation for the enrichment of key microbial taxa (e.g., *Luteolibacter*, *Arenimonas*, *Microvirga*, *Rhodoplanes*, and *Glomus*). Meanwhile, freeze-thawing inhibits the abundance of freeze-sensitive taxa such as *Flavobacterium* and *Chryseobacterium* by altering soil physical properties (e.g., pore structure destruction and reduced aeration; Li et al., 2017), while the effects on cold-tolerant taxa like *Luteolibacter* and *Arenimonas* can be disregarded. For genera such as *Microvirga* and *Rhodoplanes*, the nutrient release from straw decomposition offset the potential adverse effects of freeze-thawing, leading to increased abundance (Guo et al., 2023; Sun et al., 2017). The phenomenon that *Glomus* was a good indicator for conservation soil management was also observed in the present study, and conservation tillage causes no damage or slight disturbance to the propagules, hyphal networks and structures of *Glomus*. This condition was more conducive to the symbiosis between plants and mycorrhizal fungi (Li et al., 2024; Mhlanga et al., 2022). The above results showed that ST significantly increased the abundance of microbial taxa associated with nutrient cycling and soil fertility by regulating soil C/N cycles. Notably, the genera *Luteolibacter* and *Arenimonas* were found to be largely undisturbed by freeze-thawing and could thereby serve as potential indicator microbes for characterizing the effects of tillage practices and freeze-thawing.

### 4.3 Effects of the soil physiochemistry properties on microbial community structure

Soil freeze-thawing in winter directly affects the decomposition of straw and the transformation of nutrients by regulating microbial activity and altering soil physical structure, while different tillage practices amplify or mitigate these freeze-thawing effects through modulating straw mulch and soil disturbance. Freeze–thawing slows microbial metabolism, and combined with the low disturbance and straw mulch of ST (ST/ST-S), it promotes SOC accumulation and TN (Balla Kovács et al., 2024; Hu et al., 2025; Yang et al., 2025). In contrast, the strong disturbance treatment (RT/ST-M) enhances the mineralization of organic nitrogen due to the intensified freeze–thawing effect, thereby increasing MN (Jacek and Piotrowska Długosz, 2024). Freeze-thawing inhibits phosphorus (P) and potassium (K) mineralization and exacerbates nutrient leaching, resulting in an overall decrease in TP, AP, and AK in 2021 (Table 1). However, ST maintains higher P and K indices due to nutrient supply from straw decomposition and reduced nutrient loss associated with low soil disturbance (Liu et al., 2019). Additionally, ST-S sustains a relatively high soil pH through freeze–thaw-suppressed organic acid secretion and alkaline substance release during straw decomposition. Specifically, ST-S sustains a relatively high soil pH through freeze-thaw-suppressed organic acid secretion and the release of alkaline substances during straw decomposition (Wang et al., 2025). In summary, soil freeze-thaw processes in winter plays a crucial role as a driving factor in the decomposition of straw and the transformation of nutrients by regulating microbial activity and altering soil physical structure. Meanwhile, different tillage practices further amplify or mitigate the effects of freeze-thawing by modulating the status of straw mulch and the degree of soil disturbance.

In this study, significant correlations were observed between soil physicochemical properties and microbial community structure, aligning with existing findings that changes in EC and SOC significantly influence microbial community composition (Dong et al., 2024; Liu Z. -K. et al., 2025; Yu et al., 2022). The increase in both macro- and micronutrients- primarily resulting from straw decomposition - contributed to elevated EC and SOC levels, thereby creating favorable conditions for microbial proliferation (Han et al., 2025; Zhang M. et al., 2025). Among all measured factors, soil pH emerged as the most influential factor of microbial community structure (Figure 5). This observation is consistent with previous studies that emphasized the central role of pH in shaping microbial ecology (Jiao and Lu, 2020; Liu Z. -K. et al., 2025). Meanwhile, a decline in soil pH was associated with reduced diversity, particularly microbial taxa with narrower niche breadths (Gu et al., 2024), suggesting pH sensitivity among specialized microbial groups. Moreover, soil P exhibited significant correlations with both overall microbial community structure and the taxa of *Arenimonas* and *Luteolibacter*. This relationship may be attributed to P release from decomposing straw which likely alleviated P limitation in the soil (Wang et al., 2025). As an essential nutrient, P directly supports bacterial growth and can substantially influence microbial community structure (Zhang et al., 2022). Notably, while prior studies have often highlighted the heightened sensitivity of bacterial communities to environmental fluctuations, our findings suggest that fungal communities were more responsive to changes in environmental factors, particularly under freeze-thaw conditions (Chen et al., 2024; Zhang N. et al., 2024). This discrepancy could stem from the winter freeze-thawing cycles characteristics of the study region, which may modulate the effects of tillage practices on microbial structure. Fungi, known for their rapid physiological responses to temperature shifts, appear especially more sensitive to such seasonal disturbances (Zong et al., 2023).

### 4.4 Effects of strip tillage on soil microbial community function

In this study, ST significantly increased the AAs of functional microbial taxa associated with nitrification, aerobic nitrite oxidation, nitrate denitrification, nitrite denitrification, anoxygenic photoautotrophic S oxidation, and ectomycorrhizal after freeze-thaw cycles (Figure 7). The increased abundance of these microbial functions is closely associated with the favorable soil environment shaped by ST. Freeze-thawing alternation causes soil pore structure disruption and aeration fluctuations, thereby directly interfering with the metabolic activity of N cycling microorganisms (Su et al., 2024). In contrast, ST with low disturbance can effectively maintain the stability of soil aggregates after freeze-thawing (Keshavarz Afshar et al., 2022). Meanwhile, straw mulch continuously supplements organic nitrogen sources, ensuring that the TN content in the ST treatments remains stable after freeze-thawing and is significantly higher than that in the RT treatment (Table 1), thus providing sustained nutrient support for N cycling functional microorganisms (Han et al., 2026; He et al., 2019). Previous studies have showed that members of the phylum Proteobacteria, notably *Rhodoplanes*, are actively involved in N cycling processes (Cui et al., 2017) and serve as key taxa in regulating denitrification under varying tillage practices (Dong J et al., 2023; Lv et al., 2024). Furthermore, *Flavobacterium*, a root-associated genus within Bacteroidetes, has been shown to possess denitrification-related genes (Abdelhamed et al., 2021), highlighting it’s potential functional contribution to soil N dynamics. In addition to nitrogen processes, cover cropping under NT has been associated with increased levels of extractable S, particularly in the topsoil, thereby enhancing S cycling at the soil surface (Filippi et al., 2024; Shambhavi et al., 2025). In this present study, ST was also found to significantly promote S oxidation function, which may be related to straw mulch, further supporting its role in nutrient cycling enhancement. Moreover, ectomycorrhizal fungi play an important role in plant–soil interactions by inducing the synthesis of endogenous hormones (such as auxins and cytochromes) and secondary metabolites that regulate phytohormonal and metabolic balance (Yu and Liu, 2002). Their well-developed mycelial networks, such as the Hartig net, also contribute to soil structure by binding soil particles and promoting macroaggregate formation (Zheng et al., 2014). Furthermore, the low disturbance characteristic of ST precisely preserves the integrity of the mycelial network, preventing hyphal damage caused by soil structure fragmentation during freeze–thawing. In summary, ST provides a favorable habitat for functional microorganisms by regulating soil physicochemical properties after freeze–thawing, thereby enhancing key ecological functions such as N and S cycling.

Due to the inherent limitations of this study, such as the lack of multi-point long-term field comparative research and that the analysis of microbial functional differences is based on predicted results rather than actual direct measurements, the generalizability and applicability of the conclusions of this study may be limited.

## 5 Conclusion

This study revealed a significant interactive effect between freeze–thawing and tillage practices. Meanwhile, freeze-thawing exerted a more prominent impact on microbial community structure, narrowing the differences in fungal communities between RT and ST, which indicated that fungal communities could serve as sensitive bioindicators under freeze-thawing conditions. In terms of microbial community characteristics, we identified *Luteolibacter* and *Arenimonas* as indicator taxa for ST, with their key trait being significant resistance to freeze-thawing. Their stable presence provided a microbial foundation for the sustained stability of soil nutrient cycling functions under ST. Furthermore, ST constructed a freeze-thawing buffering microenvironment through low soil disturbance and continuous C and N sources derived from straw mulching. This microenvironment enriched functional microorganisms associated with N cycling, S oxidation, and ectomycorrhizal, thereby enhancing the key ecological functions of black soil. Thus, this study provides a new theoretical basis for adaptive agricultural management strategies based on microbial mechanisms in response to climate fluctuations.

## Data Availability

The datasets presented in this study can be found in online repositories. The names of the repository/repositories and accession number(s) can be found at: https://nmdc.cn/, NMDC40092791-400928814.

## Footnotes

1
http://huttenhower.sph.harvard.edu/galaxy
